# Chuanxiong improves angiogenesis via the PI3K/AKT/Ras/MAPK pathway based on network pharmacology and DESI-MSI metabolomics

**DOI:** 10.3389/fphar.2023.1135264

**Published:** 2023-05-05

**Authors:** Xue-hao Cheng, Xue-xin Yang, He-rong Cui, Bei-bei Zhang, Ke-dian Chen, Xiao-yun Yang, Jing-yi Jiao, Ya-wen Du, Qi Zhang, Jia-xin Zheng, Wei Xie, Fei-fei Li, Hai-min Lei

**Affiliations:** ^1^ School of Chinese Materia Medica, Beijing University of Chinese Medicine, Beijing, China; ^2^ Waters Technology Co., Ltd., Beijing, China; ^3^ School of Life Sciences, Beijing University of Chinese Medicine, Beijing, China

**Keywords:** Chuanxiong, angiogenesis, network pharmacology, DESI-MSI metabolomics, mechanism

## Abstract

**Introduction:** Chuanxiong, a traditional Chinese medicine, has been proved to treat a variety of cardiovascular and cerebrovascular diseases by promoting angiogenesis. However, the mechanisms of Chuanxiong’s pro-angiogenesis is currently unknown. This study aimed to uncover the effect and mechanisms of Chuanxiong promoting angiogenesis *in vivo* and *in vitro*.

**Methods:** First, potential targets were predicted by network pharmacology analysis, and PPI network was established and the pathways were enriched. Then, the chorioallantoic membrane test on quails was applied to assess the proangiogenic effects *in vivo*. As well, to evaluate the effects *in vitro*, real-time PCR, western blot analysis, the scratch test, and the tube formation experiment were used. Subsequently, the major metabolic pathways were analyzed using non-targeted metabolomics.

**Results:** As a result of network pharmacological analysis, 51 collective targets of Chuanxiong and angiogenesis were identified, which are mainly associated with PI3K/AKT/Ras/MAPK pathway. And the biological verification results showed that Chuanxiong could increase the vessel numbers and vessel area in qCAM models. Meanwhile, Chuanxiong contributed to HUVEC proliferation, tube formation, migration, by encouraging scratch healing rates and boosting tube branch points. In addition, the levels of VEGFR2, MAPK and PI3K were elevated compared to the control group. The western blot analysis also confirmed Chuanxiong could promote an increase in AKT, FOXO1 and Ras. Furtheremore, metabolomic results showed that the proangiogenic effect of Chuanxiong is associated with glycine, serine and threonine metabolism.

**Discussion:** In conclusion, this study clarified that Chuanxiong could promote angiogenesis *in vivo* and *in vitro* via regulating PI3K/AKT/Ras/MAPK pathway.

## 1 Introduction

Angiogenesis is an important physiological process involved in almost all diseases, and it specifically plays an important role in the treatment of myocardial infarction, stroke, cerebral embolism, and other ischemic diseases (Apte et al., 2019). Therefore, targeted stimulation of angiogenesis-related pathways will provide a new treatment option for these diseases. At present, the main techniques for therapeutic angiogenesis are as follows: up-regulating the expression of related cell growth factors (Miyagawa et al., 2002) and promoting the proliferation and migration of vascular endothelial cells (Duan et al., 2019). However, traditional drugs for angiogenic diseases easily cause dizziness, hypotension, and bradycardia. With low prices, few adverse reactions, and almost no drug resistance, traditional Chinese medicine has gradually become a new research hotspot in the treatment of angiogenic diseases.

The dry root and rhizome of *Ligusticum chuanxiong* Hort., is a kind of traditional Chinese medicine named Chuanxiong. Numerous pharmacological investigations suggest that it has an impact on the circulatory, digestive, and central nervous systems. So a wide range of cardiovascular and cerebrovascular diseases were cured by Chuanxiong, including hypertension, cerebral infarctions, angina pectoris, and coronary heart disease (Ran et al., 2011). According to the ancient Chinese medicine book “Shen Nong’s Herbal,” Chuanxiong has a pungent taste and is warm in nature, and its material distribution is the liver and heart. It could promote blood and qi circulation and relieve the pain. According to the Chinese medicine classic “Compendium of Materia Medica,” Chuanxiong could treat blood deficiency. According to the “Chinese Pharmacopoeia,” Chuanxiong was used to treat chest stuffiness and pains, headache, and angina pectoris. Modern studies have shown that a critical component of the treatment of Chuanxiong’s cardiovascular and cerebrovascular illnesses is the proangiogenic effects (Shi et al., 2019), but the mechanism is still unclear.

In this study, we used a network pharmacology approach to identify the potential targets and pathways of Chuanxiong in the effect of angiogenesis. The integration of metabolomics and network pharmacology results showed that Chuanxiong improves angiogenesis via the PI3K/AKT/Ras/MAPK pathway. Furthermore, to investigate potential underlying mechanisms via which Chuanxiong transmitted the promotion effects on HUVEC proliferation and angiogenesis, we performed both *in vitro* and *in vivo* tests. In addition, we used Western blotting and RT-PCR to verify that Chuanxiong could increase the expression of key proteins in PI3K/AKT/RAS/MAPK pathways. In conclusion, our results demonstrated that Chuanxiong could promote angiogenesis via PI3K/AKT/RAS/MAPK pathways, which paves the way for the potential treatment of Chuanxiong for the various vascular diseases.

## 2 Materials and methods

### 2.1 Network pharmacology analysis

First, the active ingredients of Chuanxiong were screened from the TCMSP database (Ru et al., 2014) (https://www.tcmsp-e.com/). To find the active ingredients, the DL value should be less than 0.18, and the OB value should be less than 30%. Second, each ingredient’s SMILE and SDF files were obtained from the PubChem database (Kim et al., 2019) (https://pubchem.ncbi.nlm.nih.gov/). Third, the targets associated with Chuanxiong were ascertained from SwissTargetPrediction. The targets related to Chuanxiong were demonstrated in SwissTargetPrediction (Daina et al., 2019) (http://www.swisstargetprediction.ch/). We chose the targets with probability values less than 0. Using “angiogenesis” as the search term, we used the DrugBank (https://www.drugbank.com/) to extract angiogenesis-related targets. The obtained Chuanxiong targets and disease targets were topologically calculated by Cytoscape 3.7.1, and the “drug–ingredient–disease–target” network was performed. Based on Chuanxiong targets for angiogenesis, protein–protein interactions (PPIs) were gathered from STRING (https://cn.string-db.org/cgi/input.pl).The network of PPI was constructed, and the topological features of the network were analyzed to screen out the core targets that play a crucial role in the PPI network. We used Metascape to perform GO enrichment and KEGG pathway enrichment analyses of target genes, screening results with a *p*-value of 0.01, a minimum count of 3, and an enrichment factor of 1.5%.

### 2.2 Chemicals and reagents

Quail hatching embryos were obtained from the Qingfeng Aichongyuan livestock farm, Shandong Rizhao, China. HUVEC cell lines were obtained from the Chinese Academy of Medical Sciences. Dulbecco’s modified Eagle’s medium (DMEM), heat-inactivated fetal bovine serum (FBS), streptomycin, and penicillin were obtained from Thermo Scientific (Waltham, United States). Power SYBR Green PCR Master Mix reagents and gel embedding medium were obtained from Sigma-Aldrich (St. Louis, Missouri, United States. Methanol (≥99.9%), pyridine (≥99%), and acetonitrile (HPLC grade) were obtained from Beijing Chemical Plant Co. Ltd. (Beijing, China). Compounds 2-chloro-L-phenylalanine (≥98%) and N, O-Bis (trimethylsilyl) trifluoroacetamide with 1% trimethylchlorosilane (BSTFA +1% TMCS) were obtained from Shyuanye Biochemical Co., Ltd. (Shanghai, China; CAS number 103616-89-3, 25,561-30-2). Double-distilled water was purified from the Millipore water purification system (Millipore, Bedford, MA, United States). Methoxyamine hydrochloride (≥98%) and thiazolyl blue tetrazolium bromide (MTT, ≥98%) were purchased from Beijing Biorigin Biotechnology Co., Ltd. (Beijing, China). Tetrahydropalmatine (CAS No.2934-97-6,99.6%) and tert-butylhydroperoxide (CAS No.75.91.2, 99.6%) were purchased from Innochem (Beijing, China).

### 2.3 Preparation of extracts from Chuanxiong for qCAM and cell assays

The Chuanxiong decoction was purchased from Beijing Tong Ren Tang (Beijing, China). The Chuanxiong decoction was pulverized into powder using a disintegrator and sieved through a 24-mesh sieve. Then, 60 g of Chuanxiong powder was extracted with the 10-fold volume of deionized water for 60 min and refluxed four times (1 h/reflux). The filtrate is combined and used for lyophilization. The Chuanxiong extract was stored as a lyophilized powder at −20°C after 3 days of lyophilization.

### 2.4 Quail chick chorioallantoic membrane (qCAM) assay

Quail eggs (10 ± 2 g; 10/group) were incubated in an egg incubator under specific pathogen-free conditions, such as maintaining at 37°C and 60% humidity. The eggs were separated into five groups at random, namely, negative control, positive control, low dose (1 mg/mL), medium dose (2 mg/mL), and high dose (4 mg/mL), comprising 10 eggs per group. After 7 days of incubation, a window was opened on the egg shell, the medicine (1mL/egg) is injected into the egg through the window, and then, the eggs were put back in the incubator after the window was taped shut. After 48 h of treatment and subsequent incubation at 37°C, the *in vivo* system of vascular microcirculation was observed in the embryos by optical coherence tomography (OPTPPRORE MICRO-VCCULTIM, Beijing HealthOlight Technology Co., Ltd., China). Then, the CAMs were excised, unfolded, and photographed. The pictures were analyzed on the MATLAB platform.

### 2.5 RNA isolation and real-time PCR analysis

The TRIzol Reagent RA First Strand cDNA Synthesis Kit and an ABI Prism 7,500 sequence detection kit were used to isolate the total RNA from HUVEC samples. Life Technologies (Invitrogen; Thermo Fisher Scientific, Inc., MA United States) provided the specific VEGFR2, PI3K, and MAPK primers. The primer sequences are as follows: VEGFR2 primers were forward, 5′- AGC​ATA​GAC​AGC​CCT​TTG​GT -3′ and reverse 5′- CAC​AAT​CTC​TGC​TGG​TGC​AA -3′; PI3K primers were forward, 5′-ACT​GCC​GAG​AGA​TTT​TCC​CAC -3′ and reverse, 5′- TCA​CTC​ATC​TGT​CGC​AGG​CA -3′; and MAPK primers were forward, 5′- GAG​AGA​TGT​CTA​CAT​TGT​GCA​GGA​C -3′ and reverse, 5′- AAT​CTT​AAG​GTC​GCA​GGT​GGT​G -3’ (reverse primer). We used human GAPDH as an internal reference.

### 2.6 Cell viability assay

HUVECs were cultivated in Dulbecco's modified Eagle’s medium supplemented with 15% fetal bovine serum (FBS), 100 U/mL penicillin, and 100 g/mL streptomycin. In addition, 37°C, 5% CO_2_, and 100% humidity were the conditions used to sustain the cultures. When the cells reached 70%–80% confluence, HUVECs were digested and centrifuged. Then, one 96-well plate of HUVECs was seeded for 24 h at 37°C before it was added for an additional 24 h of incubation. A Bio-Rad 550 spectrophotometer plate reader was used to detect the optical density at 550 nm (Bio-Rad, CA, United States).

### 2.7 Wound scratch assay

HUVECs were cultured in 24-well plates until forming a monolayer. A clean scratch was made down the middle of the cell using a sterile pipette tip. Images of the same location were photographed using a light microscope (@10X, Nikon Eclipse TiE, Japan). Using Image-Pro Plus (version 5.0, National Institutes of Health, United States), we calculated the cell migration distance.

### 2.8 Tube formation assay

An assay for the closure of scratch wounds was used to investigate cell migration. In a 96-well plate, HUVECs were grown at a density of 1 × 10^4^ cells/cm^2^ with a basement membrane matrix (Corning Matrigel, CAS number 356234) until the cell confluence reached 100%, and then, Chuanxiong was added for additional 6 h. Light microscopy (@10X, Nikon Eclipse TiE, Japan) and Image-Pro Plus (version 5.0, National Institutes of Health, United States) was used to measure the length of the tubes and capture pictures of the tube networks.

### 2.9 GC-MS-based metabolomics analysis for qCAM samples

#### 2.9.1 Quail sample handling

In an optimal cutting temperature (OCT) compound, quail samples were solidified at 40°C. The samples were constantly sliced with a thickness of 15 μm. Then, the DESI-MSI system was used to scan them.

#### 2.9.2 Mass spectrometry conditions

The scan area’s dimensions (X, Y, and outline) were measured and scanned in accordance with the sections using the DESI and SYNAPT HDMS G2-Si system (Waters, MA, United States). On the scanning platform, the sample slices were fixed. The scan’s parameters are as follows. Vertical displacement was set as 0 mm, scan line spacing was set as 0.15 mm, and the delayed start was set as 6 s. The scan speed was set as 0.1 mm/s. For mass spectrometry, a desorption electrospray ionization source (DESI) was used. The voltage of the electrospray was 5.0 kV. The spray solution utilized was 90% methanol, and the flow rate was set at 1.5 L/min. The nebulizer’s setting was 100 pis. The distance between pixels was 50–200 m. A mass range of 80–1,000 m/z was chosen.

#### 2.9.3 Data processing and pattern recognition analysis

Data preprocessing was carried out using MarkerLynx (Waters, MA, United States). With the aid of MetaboAnalyst 5.0 (Quebec, Canada, https://www.metaboanalyst.ca/home.xhtml), data were analyzed and the metabolic pathway was obtained. The findings of the t-test (*p* <0.05), the ANOVA (*p*< 0.05), and the fold change value (>1.5) were used to filter out significant differences. Using the online METLIN database, variables with substantial changes were identified as prospective biomarkers for provisional identification. MetaboAnalyst 5.0 was used to conduct pathway analysis based on the discovered biomarkers.

### 2.10 Western blotting

The cell samples from the cell lines were extracted using lysis buffer ultrasonically in ice. After 10 min of centrifugation at 4°C at a speed of 12,000 rpm, the supernatant was collected. Then, the concentration was measured using the BCA kit (Beyotime) according to the manufacturer’s instructions. SDS-PAGE gel electrophoresis was used to separate the proteins, and they were then transferred to a polyvinylidene fluoride film. After blocking in 5% BSA for 1 h, the membranes were treated with the corresponding primary antibodies (1:1,000) at 4°C overnight. After washing, the membranes were then incubated with the corresponding alkaline phosphatase-conjugated secondary antibodies (1:1,000) for 1 h. The Tanon 4200 SF Chemiluminescent Imaging System (Shanghai Tanon, Shanghai, China) was used to expose the membranes.

### 2.11 Statistical analysis

Data were analyzed by SPSS 16.0 software (Chicago, IL, United States). The data are displayed as mean ± standard deviation. In order to compare different groups, one-way analysis (ANOVA) of variance was used. Each two groups were contrasted using the independent t-test. These data discrepancies were deemed statistically significant when the differences reached a *p*-value of 0.05.

## 3 Results

### 3.1 Chuanxiong promotes angiogenesis via multiple targets according to network analysis

**FIGURE 1:**
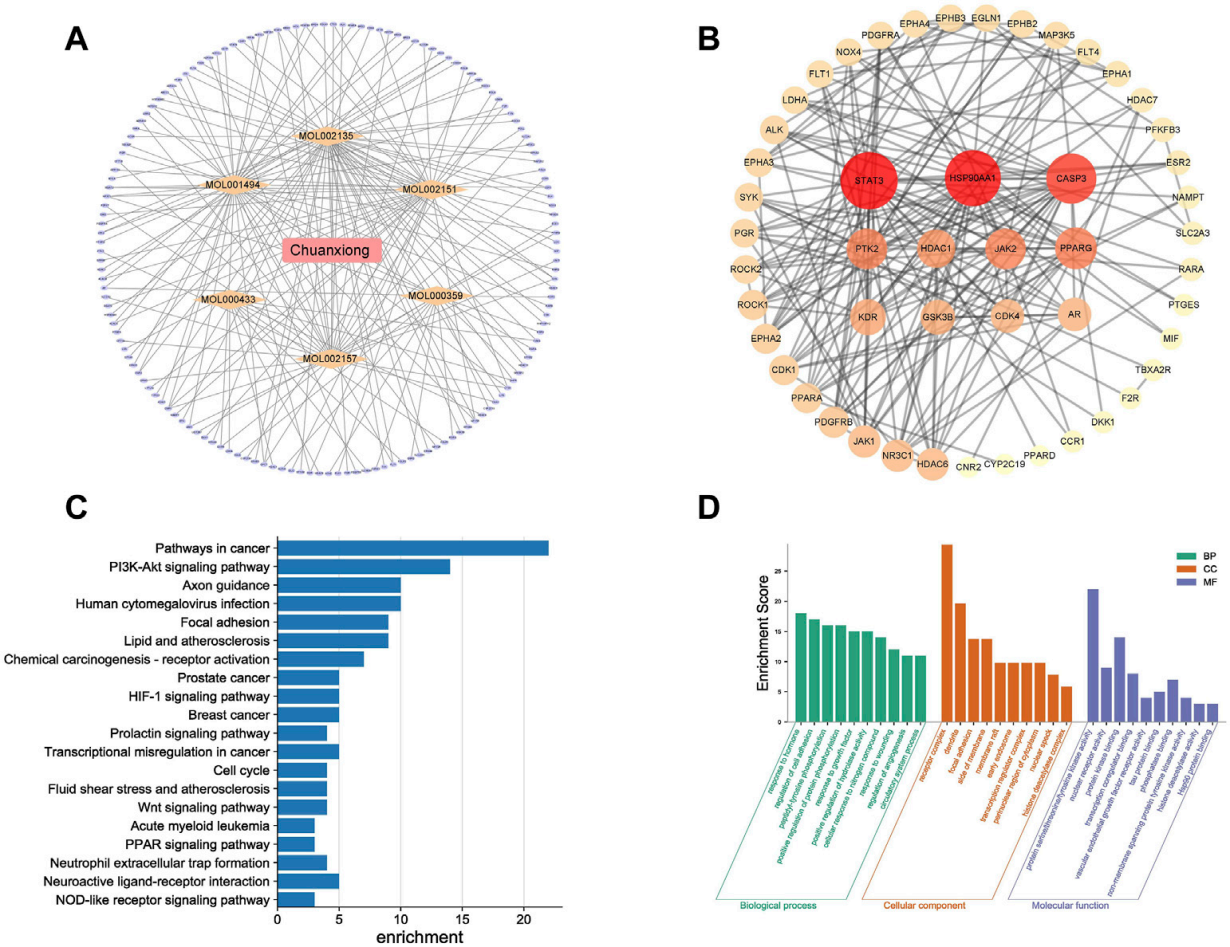
Results of network pharmacology for investigating potential mechanisms of Chuanxiong in angiogenesis: **(A)** Drug–ingredient–target–pathway network. The orange diamonds are active components of Chuanxiong, and the purple ellipse nodes are the related targets: **(B)** Target protein interaction network of Chuanxiong from IPA; deeper colors represent higher node connectivity. **(C)** Top 20 pathway terms enriched by the KEGG database. **(D)** Enriched top 10 GO pathways in the biological process, cellular component terms, and molecular function terms.

The TCMSP database had 189 active components of Chuanxiong in total, and seven active ingredients satisfied the criteria of OB value ≥30% and DL value ≥0.18. Then, 627 targets were obtained by SwissTargetPrediction. A total of 4,975 targets related to angiogenesis were obtained from the DrugBank. The intersection of component and angiogenesis targets yielded 51 common targets, and the drug–ingredient–disease–target network was constructed (Figure 1A).

The target interaction network graph is obtained with the confidence score set at 0.4 (Figure 1B). The nodes represent differentially expressed proteins, and the edges represent their interactions. There were 51 nodes and 203 edges in the network. Seven nodes with degrees higher than or equal to the median (degree = 8) of all nodes were selected as the core components and core targets of Chuanxiong in angiogenesis, including PPARG, HDAC1, CDK4, JAK2, HSP90AA1, CASP3, and SYAT3. These targets might be crucial to Chuanxiong’s ability to affect angiogenesis. A total of 44 cellular component terms, 81 molecular function terms, and 439 biological process terms were significantly enriched by GO enrichment analysis. The top 10 enriched pathways with significant differences were selected to construct a bubble chart (Figure 1C). Similarly, according to the *p*-value, the top 20 pathways in the KEGG enrichment study are shown (Figure 1D).

### 3.2 Chuanxiong leads to increased angiogenesis in a dose-dependent manner in qCAM

**FIGURE 2:**
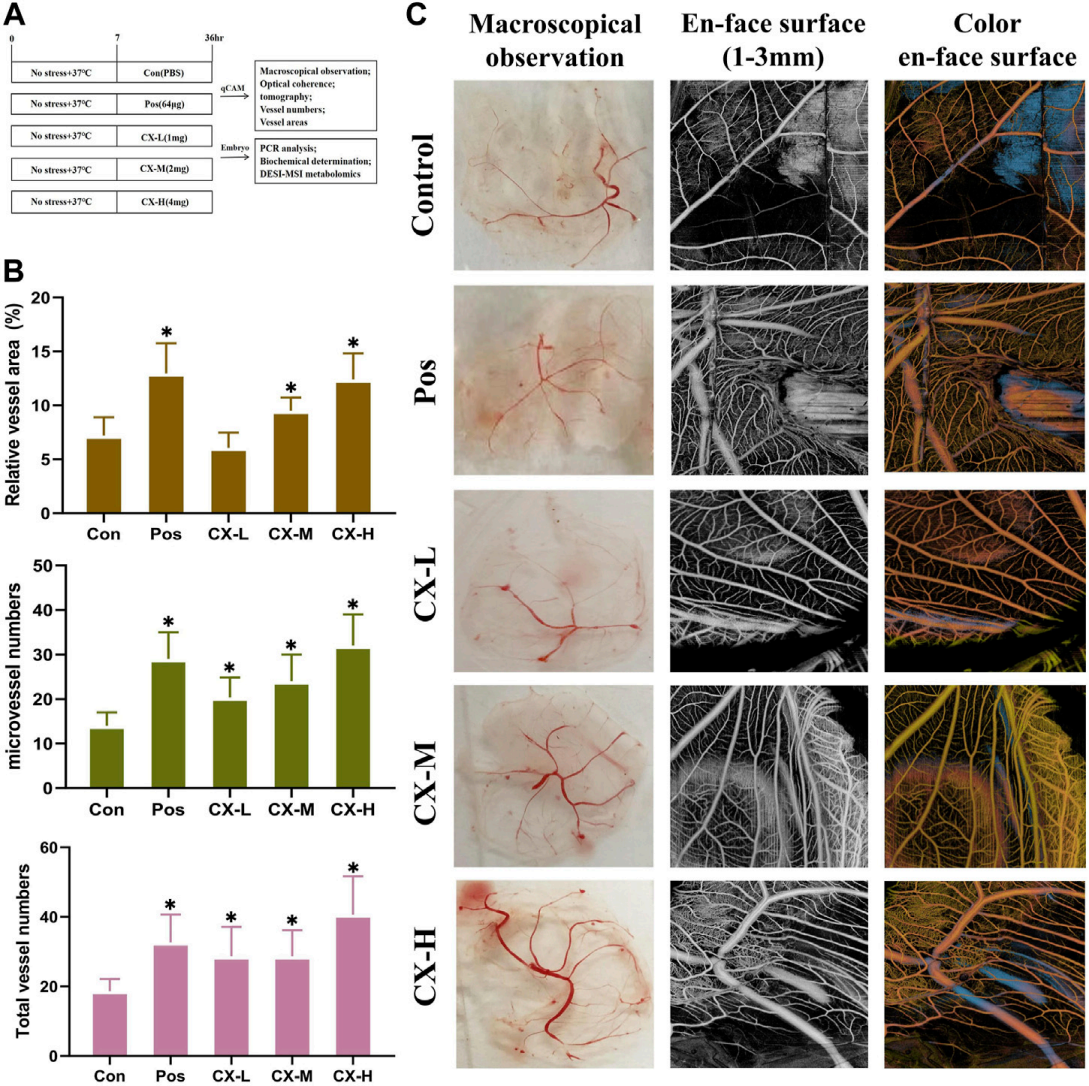
Chuanxiong stimulated angiogenesis of qCAM in a dose-dependent manner. **(A)** Experimental design’s schematic diagram. Con, the control group treated with PBS; Gas, the positive control group treated with tetrahydropalmatine (64 μg); GX-L,M,H, the groups treated with different doses (1,2,4 mg) of Chuanxiong, respectively. **(B)** Relative vessel area, microvessel numbers, and total vessel numbers of qCAM samples in the respective groups. **(C)** Pictures of macroscopical observation and optical coherence tomography angiography of qCAM. The most typical fields are displayed. The results of each experiment are shown as the mean ± S. D after being carried out three times. *p < 0.05, significantly different from the model group.

Using qCAM tests, Chuanxiong’s effectiveness in inhibiting angiogenesis was assessed (Figure 2A), and Pos was selected as the positive control according to previous studies. A measure of 1, 2, and 4 mg/mL were determined as different doses based on the previous dose screening results. Compared to the control group, the vessel numbers and vessel area significantly (*p* < 0.05) increased in the medium- and high-dose group (Figure 2B). It indicated that Chuanxiong has a notable dose-dependent proangiogenic effect. Additionally, the macroscopical results and the optical coherence tomography findings, which examined the vascular microcirculation of qCAMs *in vivo*, were in agreement (Figure 2C).

### 3.3 Chuanxiong alleviates injury induced by t-BHP *in vitro*

**FIGURE 3:**
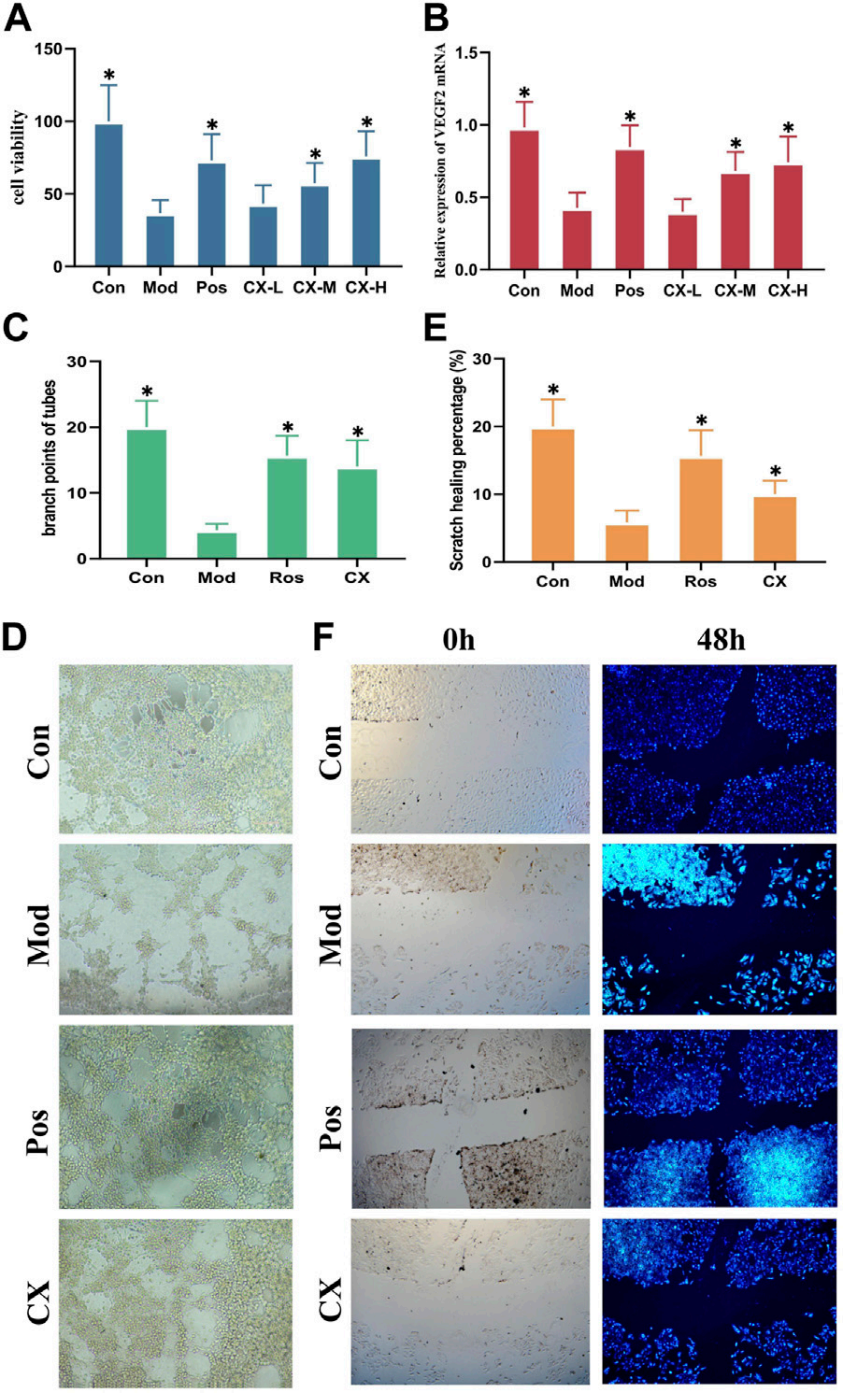
Chuanxiong promoted angiogenesis of HUVECs in a dose-dependent manner. **(A)** Viability of HUVEC cells treated with Chuanxiong or PBS after t-BHP was damaged. **(B)** mRNA levels of VEGFR2 in different groups of HUVECs. **(C, D)** Outcomes of tube formation in HUVECs treated with PBS or medications after 6 h are displayed (100×). **(E,F)** Findings of wound scratch assays of HUVECs treated with PBS or medications are displayed after 48 h (100×). Con, the control group treated with PBS; Mod, the model group treated with 5.5 μM t-BHP for 4 h; Pos, the positive control group treated with tetrahydropalmatine (40 μM); CX-L,M,H, the groups treated with different doses (25 µg, 50 μg, 100 µg) of Chuanxiong. The most representative fields are displayed. * represents *p* < 0.05 compared with the control group. The significance of differences was evaluated with ANOVA with the *post hoc* test. The results of each experiment are shown as the mean ± S. D after being carried out three times.*p < 0.05, significantly different from the model group.

On the basis of the proliferation assays of HUVECs, the effects of Chuanxiong on angiogenesis were evaluated. The administration concentrations chosen were 25 µg, 50 μg, and 100 µg . Based on the previous report, Pos (40 µM) was chosen as the positive control. The survival rate of HUVEC was represented by MTT assays after 4 h of exposure to 5.5 μM tert-butylhydroperoxide (t-BHP), according to the preliminary assay that we performed (Cui et al., 2021). The results of MTT assays showed that middle-dose and high-dose groups could considerably (p<0.05) restore cell viability in t-BHP-damaged cells, protecting them against further harm (Figure 3A). Furthermore, VEGFR2 messenger RNA (mRNA) expression levels of the gene were measured by quantitative real-time PCR. The result showed that the messenger RNA (mRNA) expression levels in middle-dose and high-dose groups were significantly (p<0.05) higher than those of models, and these changes were dose-dependent (Figure 3B).

The tube formation and wound scratch assays of HUVECs were used to further evaluate the effects on angiogenesis of Chuanxiong *in vitro*. The model condition and positive control stayed together with the proliferation assays. As shown by the results, Chuanxiong could promote scratch healing percentages and increase the branch points of tubes (Figures 3C–F).

### 3.4 Chuanxiong-regulated altered metabolic pathways are revealed by untargeted metabolomics

**FIGURE 4:**
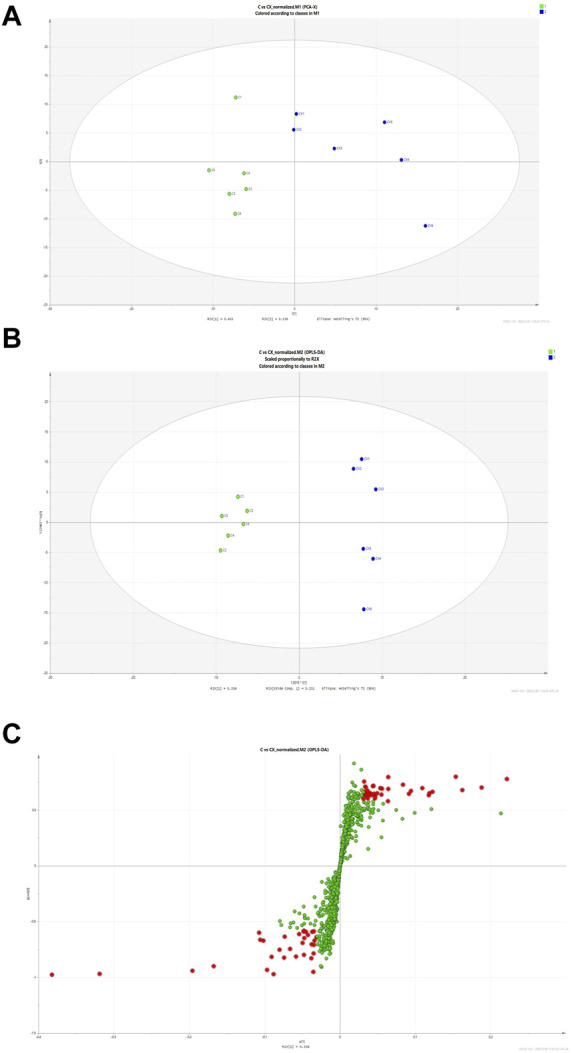
Discriminatory metabolic components and pathways regulated by Chuanxiong. **(A)** The score plots of control (C group) and Chuanxiong (E group) from PCA in the ESI + mode for PC1 *versus* PC2. QC indicates the quality control group. **(B)** OPLS-DA analysis of the data derived from the ESI + mode. OPLS-DA score plots for the pair-wise comparisons between the control and Chuanxiong groups. **(C)** S-plot of the OPLS-DA model for control and Chuanxiong groups. The points in red indicate the identified biomarkers.

The results of Chuanxiong’s target prediction indicated that the main mechanism of Chuanxiong (2/5 with low *p*-value of top term) was connected to metabolic pathways. Therefore, we used DESI-MSI based on non-targeted metabolomics to explore the possible metabolic pathway of the pro-angiogenic effect of Chuanxiong. A scoring plot with R^2^Y (cum) of 0.779 and Q^2^ (cum) of 0.463 was obtained, showing that the principal component analysis (PCA) and orthogonal structure to latent structure discriminant analysis (OPLS-DA) projection had good adaptability and predictive power for the potential model (Figures 4A, B). The variables highly correlated with group separation were selected using the projection (VIP) values of the OPLS-DA model and the variable importance of the s-plot. In other words, variables with |p (corr)| ≥ 0.58 and VIP value > 1 were selected as key metabolites and identified with METLIN (Figure 4C). Thus, the results showed seven potential biomarkers were identified by analysis (Table 1). Pathway analysis was performed using MetaboAnalyst, and it was concluded that the metabolites affecting glycine, serine, and threonine metabolism in Chuanxiong were more variable (*p* < 0.05).

**TABLE 1:** Differential identified metabolites for discrimination among control and Chuanxiong groups by DESI-MSI.

Metabolite	M/z	HMDB ID	VIP	*p* (corr)
Betaine	140.0691	HMDB0000043	11.977	−0.976209
Fluciclovine	156.0429	HMDB0244302	9.97295	−0.967688
PG (22:5 (7Z,10Z,13Z,16Z,19Z)/18:0)	825.5599	HMDB0116622	1.87558	−0.812757
Heptyl ketone	227.2229	HMDB0059813	3.10336	−0.933519
Fenpropidin	274.2747	HMDB0252217	1.20221	−0.829799
6-Hydroxynon-3-enoylcarnitine	257.1389	HMDB0241752	2.7658	−0.97099
Decarbamoylneosaxitoxin	273.1295	HMDB0033663	1.1372	−0.951031

Note: The significant differences were generated from the Student’s *t*-test or Mann–Whitney *U* test when the Student’s t-test was not suitable.

### 3.5 Effects of Chuanxiong on the expression of key proteins in the angiogenesis process

**FIGURE 5:**
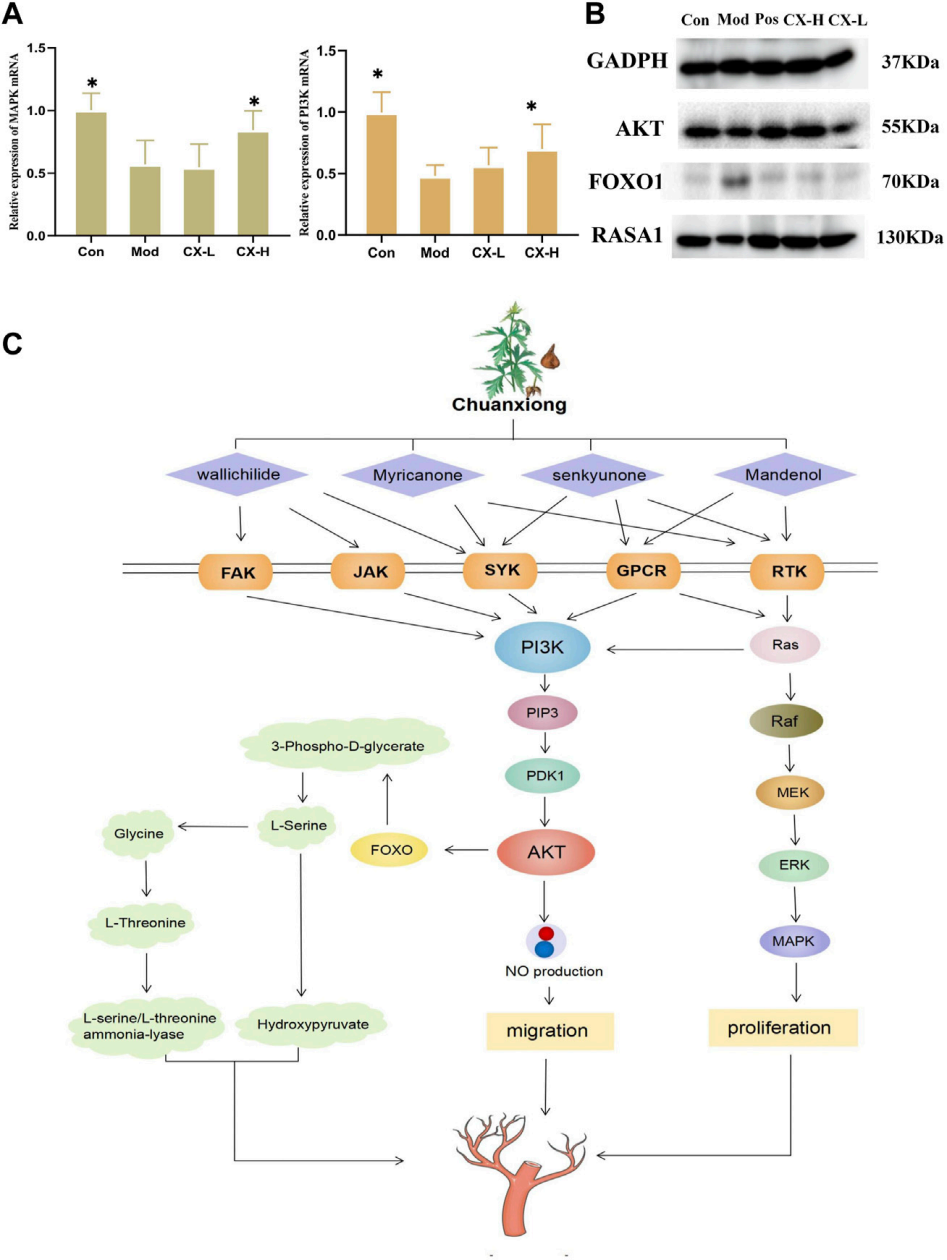
Chuanxiong promoted angiogenesis through the PI3K/Akt signaling pathway. **(A)** MAPK and PI3K levels in HUVEC cell samples, respectively. Con, the control group treated with PBS; Mod, the model group treated with 5.5 μM t-BHP for 4 h; CX-L,H, the groups treated with different doses (25 µg, 100 µg) of Chuanxiong.* represents *p* < 0.05 compared with the model group. **(B)** Protein expression of PI3K/AKT/RAS/MAPK pathway downstream targets was measured by Western blotting. **(C)** The diagram shows that Chuanxiong promotes angiogenesis by increasing PI3K expression, followed by upregulation of AKT and MAPK. These targets form a complex network of interactions and a feedback regulatory axis that when combined contribute to Chuanxiong’s effect of promoting angiogenesis.

To estimate the underlying mechanism of Chuanxiong in HUVECs under oxidative stress, the mRNA expressions of MAPK and PI3K were detected in each group. The result shows that the activity of MAPK and PI3K, two indicators of angiogenesis, was reduced with t-BHP stimulation and improved after 100 μg/mL Chuanxiong treatment (Figure 5A). In addition, as determined by Western blotting, Chuanxiong increased the expression of the AKT, FOXO1, and RASA1 after t-BHP stimulation (Figure 5B). These data demonstrate that the ability of Chuanxiong to protect against impaired angiogenesis depends on PI3K/AKT/RAS/MAPK signaling.

## 4 Discussion

According to the ancient Chinese medicine book “Shen Nong’s Herbal,” Chuanxiong has a pungent taste and is warm in nature. It could promote blood and qi circulation and relieve the pain. As a frequently prescribed drug for enhancing blood flow and reducing blood stasis, Chuanxiong is often used in combination with Baizhi and Danshen, which has a good effect on stroke, cerebral infarction, and myocardial infarction. According to our results, Chuanxiong has been shown to have considerable effects in protecting the cardiovascular system and brain tissue by preventing vascular endothelial cell death. The qCAM assay results showed Chuanxiong could increase vessel numbers and vessel area. All those aforementioned particulars indicated that Chuanxiong shows potential of promoting angiogenesis. The network results showed that the main active ingredients of Chuanxiong act on FAK, JAK, SYK, GPCR, RTK, and other receptors. These receptors can activate the PI3K/AKT/MAPK pathway, which plays a significant role in regulating angiogenesis (Mócsai et al., 2010; Zhang et al., 2020a; Yuan et al., 2021). Western blotting and RT-PCR were used to further confirm that Chuanxiong could promote angiogenesis by activating this pathway; the results showed that the expression of key proteins FOXO, RASA1, and AKT increased significantly, and cellular PI3K and MAPK levels were higher than those of control and model groups.

The phosphatidylinositol 3-kinase (PI3K) family of enzymes plays an important role in the control of cellular processes such as proliferation, apoptosis, motility, and cell growth (Koch et al., 2021). GCPRs and RTKs are upstream signals that directly interact with the regulatory subunit of PI3K to regulate PI3K activity (Fruman et al., 2017). Once active, PI3K produces (phosphatidylinositol 3,4,5-triphosphate) PIP3, which encourages the phosphorylation of AKT, which, in turn, phosphorylates a vast number of downstream substrates like eNOS to regulate angiogenesis (Revathidevi and Munirajan, 2019). At the same time, AKT can inhibit FOXO1 by reducing the expression of apoptotic genes and maintaining vascular stability (Xie et al., 2019). Ras proteins are membrane-bound small GTPases that work as molecular transducers to control cellular processes such as cell division, differentiation, migration, and death by coupling cell surface receptors to intracellular effector pathways. Ras’s GTPase activity is improved and accelerated by RASA1, a member of the RasGAP family (Zhang et al., 2020b). Ras signaling is started and activated by G-protein-coupled receptors (GPCRs) and receptor tyrosine kinases (RTKs). Ras facilitates the serine/threonine kinase (Raftranslocation)’s to the plasma membrane, where it is turned on and phosphorylated by several protein kinases (Rauen, 2013). Raf is moved to the plasma membrane by active Ras, where it is activated and phosphorylated by several protein kinases. Raf, which is active, phosphorylates and activates (mitogenic effector kinase) MEK1/2, which, in turn, phosphorylates (cytosolic signal-regulated kinase) ERK1/2, which then goes on to act on mitogen-activated protein kinase (MAPK) (Guo et al., 2020). Moreover, Ras stimulates PI3K catalytic activity by interacting with the catalytic subunit p110α and mediates a closer interaction between PI3K and the plasma membrane. Thus, RTK can activate Ras, which, in turn, activates PI3K in a p110-dependent manner (Cuesta et al., 2021).

Metabolomic analysis of qCAM models after Chuanxiong administration using DESI-MSI identified seven metabolic differentials, which were considered to be related to the metabolism of glycine, serine, and threonine. Glycine, serine, and threonine are important biosynthetic linkers that together provide precursors necessary for the synthesis of proteins, nucleic acids, and lipids, and also play important roles in enhancing protein synthesis and inhibiting protein degradation in cells, activating mTOR expression and inhibiting protein hydrolysis gene expression, all of which are essential for cell proliferation (Tsuji-Tamura et al., 2020). The metabolomics of glycine, serine, and threonine in quail chick chorioallantoic membranes increased, which indicated that Chuanxiong mainly acts on vessels.

This study identified that Chuanxiong could protect damaged HUVECs and promote angiogenesis by pharmacological tests and investigated the potential mechanisms of the proangiogenic effects of Chuanxiong through network pharmacology combined with metabolomics. In addition, we used Western blotting and RT-PCR to verify the possible pathway. In conclusion, the present study showcased that Chuanxiong promotes angiogenesis through the PI3K/AKT/Ras/MAPK pathway (Figure 5C). This study has a potential impact on the development of Chuanxiong for vascular diseases.

## Data Availability

The datasets presented in this study can be found in online repositories. The names of the repository/repositories and accession number(s) can be found in the article/supplementary material.
